# Intratendinous Ganglion Cyst of the Extensor Indicis: A Case Report

**DOI:** 10.7759/cureus.49514

**Published:** 2023-11-27

**Authors:** João Dias, Irene Pinto, Diogo Costa, Rita Sousa, Tiago Pimenta, Rita Sapage

**Affiliations:** 1 Physical Medicine and Rehabilitation, Centro Hospitalar de Trás-os-Montes e Alto Douro, Vila Real, PRT; 2 Physical Medicine and Rehabilitation, Centro Hospitalar Universitário do Porto, Porto, PRT; 3 Orthopaedics and Traumatology, Centro Hospitalar de Trás-os-Montes e Alto Douro, Vila Real, PRT

**Keywords:** tenosynovitis, intratendinous ganglion cyst, hand tumor, extensor indicis, benign tumor

## Abstract

An intratendinous ganglion cyst is a very rare benign lesion with an unknown etiology. The clinical diagnosis can be difficult as patients may have mild symptoms or impaired hand functionality. Ultrasound and magnetic resonance imaging can differentiate a ganglion cyst from other soft-tissue tumors and tumor-like lesions and provide excellent information on the location of an intratendinous lesion to schedule surgical treatment. We present a case report of a 50-year-old female diagnosed with an intratendinous ganglion cyst of the extensor indicis. She complained of right-hand swelling for three months, which was associated with pain. The US revealed an oval hypoechoic mass with cystic formation at the extensor indicis, measuring 9 x 4 mm, compatible with an intratendinous ganglion cyst. The cyst was excised by enucleation. After surgery, the patient was referred to the Department of Physical and Rehabilitation Medicine for evaluation. She started a rehabilitation programme. The patient presented a favourable clinical evolution with a return to her previous professional activity. However, six months after surgery, the cyst recurred, but with a smaller size and no associated pain.

## Introduction

Intratendinous ganglion cysts of the hand are uncommon, and only a few cases have been documented so far [1]. Extensor digitorum communis, extensor pollicis brevis, extensor pollicis longus, and extensor digiti minimi tendon were described as the most frequently found intratendinous ganglia locations [2].

Although the exact cause of these lesions is unclear, the development of cystic space may be explained by a history of acute or recurrent chronic injury, such as tendon compression against a bony prominence [3]. The ganglion cysts may develop as a result of tenosynovitis invading the tendon or as a result of mucoid degeneration of the tendon ground substance, resulting in cavities filled with viscous fluid. Despite being benign, the lesion can be locally aggressive and impair the function of the tendons and hands [4]. Intravenous cannulation may also be related to iatrogenic intratendinous ganglion cysts [5].

The existence of ganglionic cysts can be confirmed using US and MRI, which can also be used to determine the cyst's sizes and relationships with the surrounding structures. Ganglionic cysts show up as hypoechoic structures on US with septa, lobules of various sizes, and acoustic enhancement. Colour Doppler can be used for differential since ganglionic lesions typically lack vascularity. On T1 MRI images, ganglionic cysts exhibit thin enhancement in rim shape with low signal intensity, whereas high signals are visible on T2 MRI images [6].

The position, size, and symptoms of the lesion will largely determine the treatment options [6]. Intratendinous ganglia can be successfully treated with aspiration, open excision of the ganglion, or resection of the entire tendon, followed by tenodesis or tendon transfer. For the treatment of tendon pathologic entities, tenoscopy is an effective choice and requires a smaller incision than open surgery [1].

## Case presentation

A 50-year-old female, seamstress, right-hand dominant, followed at the Department of Physical Medicine and Rehabilitation for degenerative osteoarthritis of both hands. She reported a three-month history of swelling on the dorsal aspect of the right hand. She had no history of trauma or any other relevant medical conditions. The mass became gradually larger.

On clinical examination, a palpable soft-tissue mass at the dorsal aspect of the right hand was found. The movement of her fingers triggered pain on a visual analogue scale (VAS) scale of 8/10. The subcutaneous mass moved with the active excursion of the extensor indicis tendon. Manual muscle testing for the extensor indicis was graded as four out of five (active movement against gravity and resistance). The passive range of motion of the second metacarpophalangeal joint was restricted to 80º of flexion. She did not have any sensory disturbances noted.

**Figure 1:**
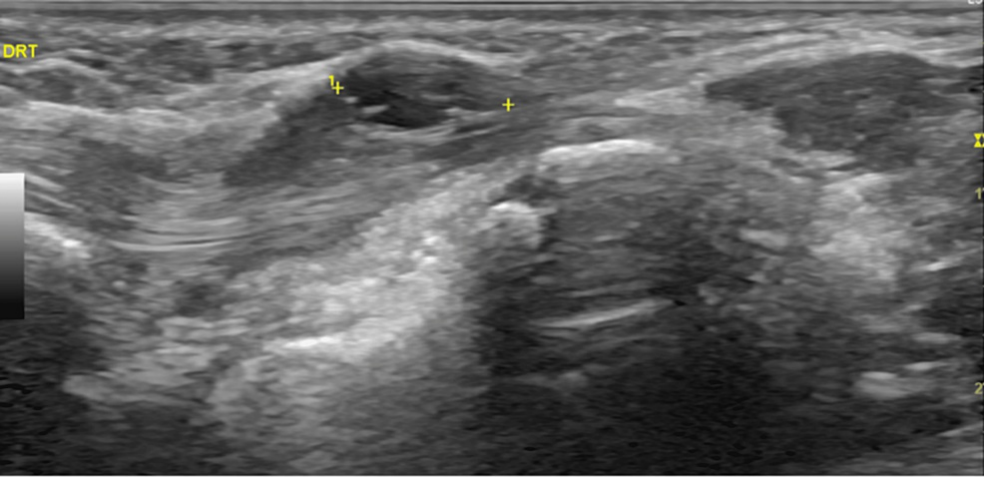
Ultrasound of intratendinous ganglio cyst at the extensor indicis

An ultrasound was performed, which showed an oval hypoechoic mass, and cystic formation at the extensor indicis, measuring 9 x 4 mm, compatible with an intratendinous ganglion cyst (Figure 1). Given the location and the high recurrence rate after aspiration, the patient was referred to the Orthopedics Department for evaluation, and surgical treatment was considered. She also started a short course of NSAIDs (seven days).

**Figure 2:**
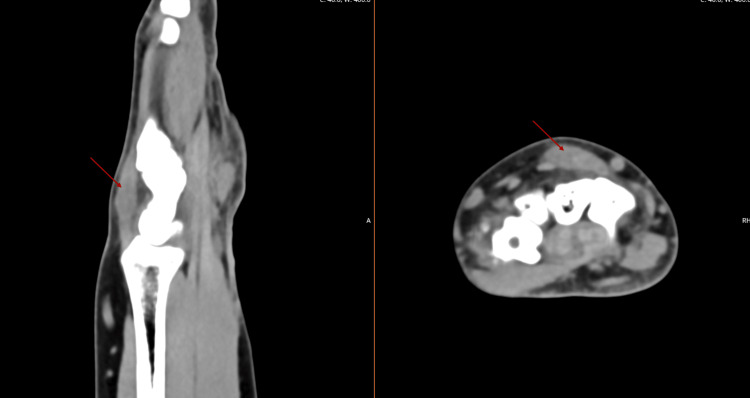
CT scan, intratendinous cyst (red arrow)

In the Orthopaedic Department, a CT scan was requested to evaluate bone structures and exclude other sources of pain (Figure 2). No relevant structural alterations were evidenced. Given the progressive clinical worsening associated with activities of daily living (ADL) limitations, the patient was proposed for surgery.

**Figure 3:**
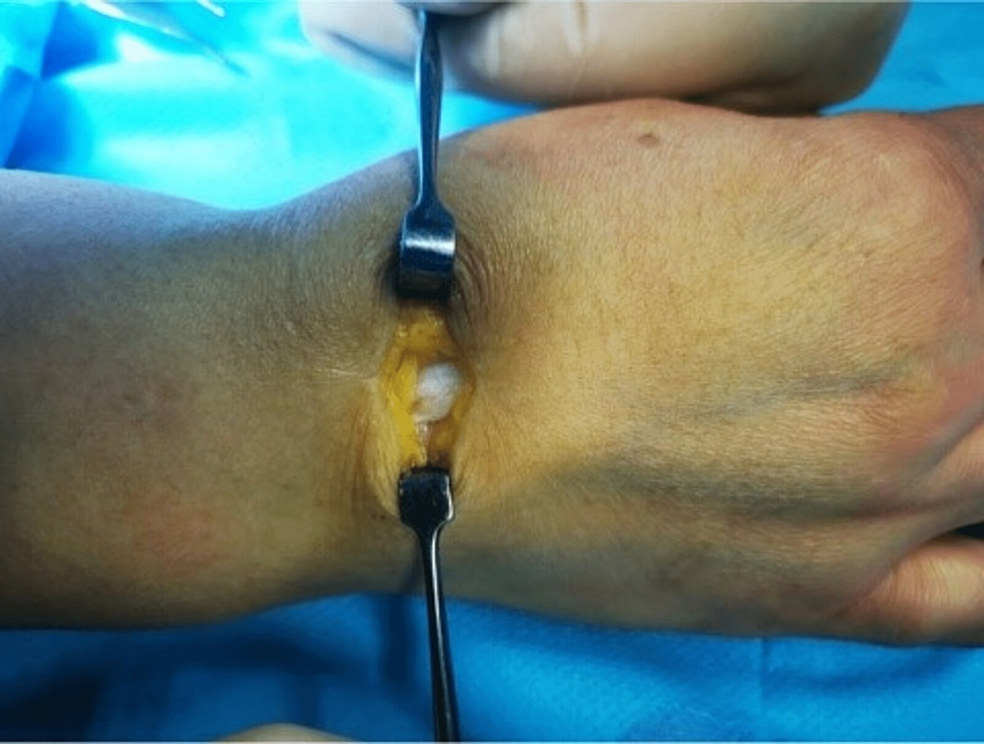
Surgical approach with identification of the tendon

**Figure 4:**
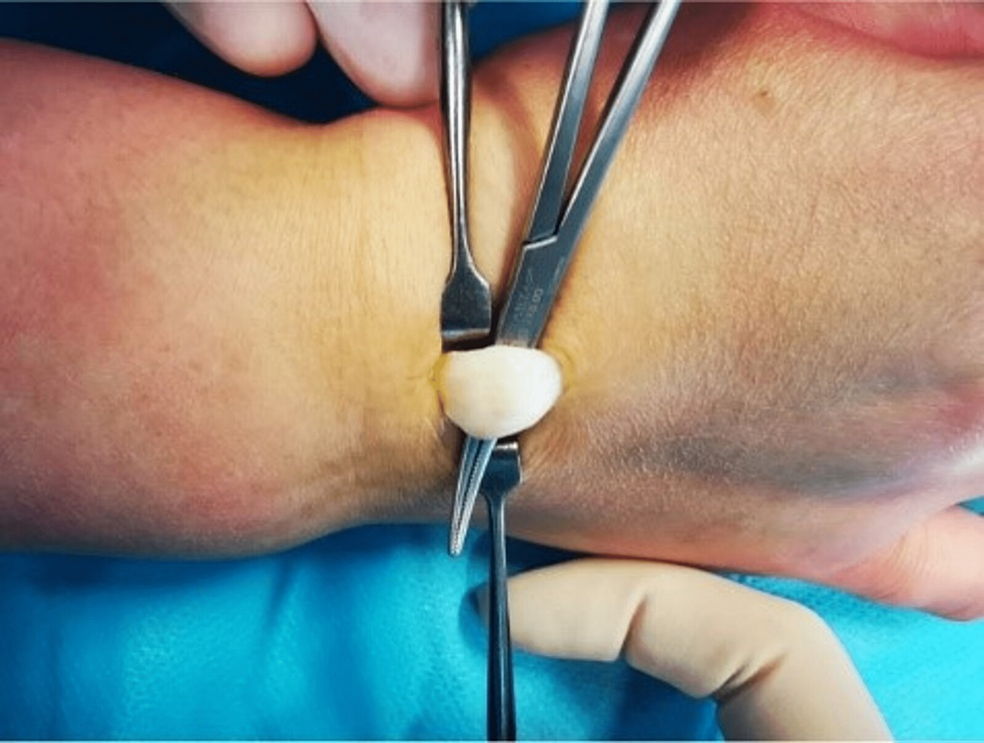
Right hand cyst excision

**Figure 5:**
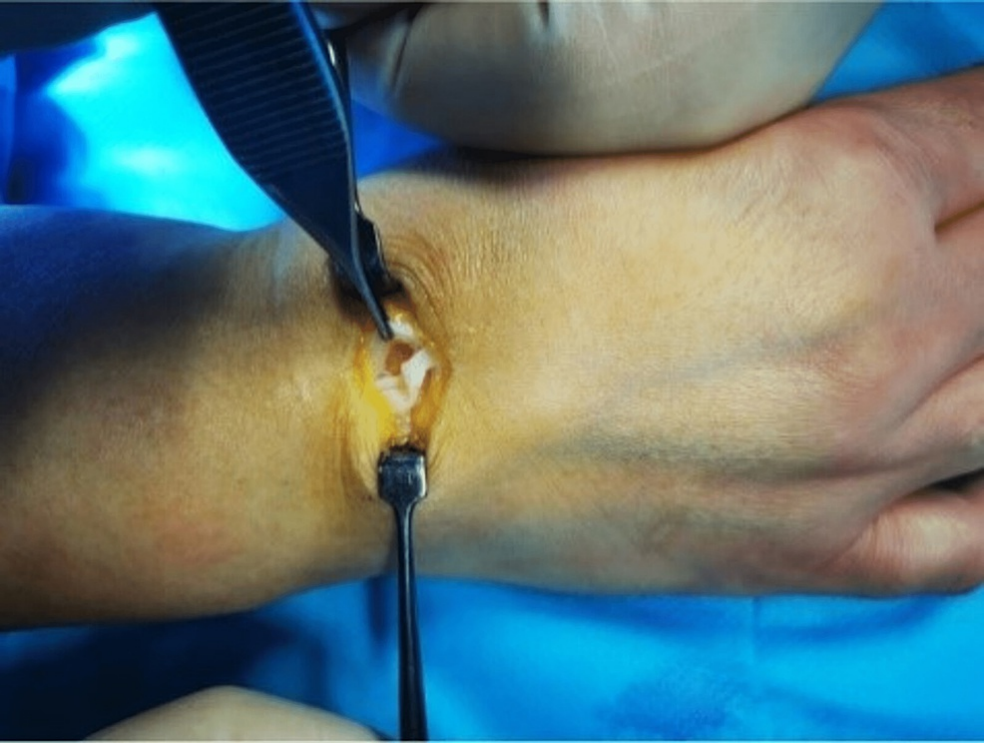
Tendon defect after the excision

Within the extensor indicis tendon, a 9 x 4 mm fusiform cyst with clear mucoid fluid was observed. Synovitis was absent. Enucleation was used to remove it through a longitudinal tendon split. The remaining cyst wall was separated from the tendon material, the tendon defect was repaired with a continuous suture (Figures 3-5), and the cyst was tubularized. The tendons were able to move freely through the full extent of the index finger following resection. A ganglion was identified by the cyst's histologic analysis.

After surgery, the patient was referred to the Department of Physical and Rehabilitation Medicine for evaluation. She started a rehabilitation programme consisting of cryotherapy, physiotherapy, and occupational therapy to promote cicatrization, tendon gliding, pain management, and range of motion muscle strengthening and training in ADL.

After completing the rehabilitation program, the patient presented a favourable clinical evolution with a return to her previous professional activity and autonomy in ADL. The physical examination showed neither pain complaints nor limitations in passive and active joint range of motion. Muscle strength was rated as five out of five according to the MRC scale.

She was discharged from Physical Medicine and Rehabilitation. Later, in a reevaluation visit to the Orthopedics Department six months after surgery, the cyst recurred. At the time of submission of this clinical case, the patient was proposed for a new surgical procedure, with excision of the cyst, which she refused.

## Discussion

Definitive diagnosis of an intratendinous ganglion cyst can be challenging due to its rarity and the unawareness of clinicians. Despite the fact that the location of intratendinous ganglion cyst formation may resemble that of typical ganglion cysts, the former are simple to differentiate by physical examination because they move in conjunction with the tendon. Granulation brought on by injuries, inflammatory or infectious tenosynovitis, and neoplasm are among the differential diagnoses [7].

A preliminary examination with US can determine whether ganglionic cysts are present. With MRI, the relationship between the lesion and the surrounding structures can be seen clearly and in much more depth [6]. In this clinical case, a CT scan was preferred over an MRI for the evaluation of possible bone tissue pathology and for the greater availability in our hospital.

As with other types of ganglion cysts, conservative treatment is acceptable in some cases. The location, size, and symptoms of the lesion largely determine the treatment choices. Cysts without associated symptoms or cosmetic issues are typically treated symptomatically and conservatively [6]. However, intratendinous ganglion cysts are associated with an increased risk of spontaneous tendon rupture, which should be considered in the therapeutic option [7].

In the case report presented, the patient presented a baseline clinical condition of osteoarthritis of the hands associated with pain with repercussions on ADL and professional activity. Nevertheless, cyst recurrence can occur either with aspiration treatment (50%) or surgical treatment (5%) [6].

## Conclusions

In this case report, the patient had osteoarthritis of the hands as a baseline clinical condition, which was accompanied by discomfort and had an impact on ADL and professional activity, with the added morbidity of the intratendinous cyst. Nevertheless, aspiration therapy (50% of cases) or surgery (5% of cases) can result in cyst recurrence. However, because IGC and related synovitis commonly damage the structure of the affected tendons, early detection is crucial. Not only can ultrasound-guided procedures harm the structure and function of the affected tendons, but they may also necessitate surgical excision.
